# Developmentally Stratified Integrated Preventive Strategies for Pediatric Oral Health: A Systematic Review with Healthcare Implications

**DOI:** 10.3390/healthcare14142074

**Published:** 2026-07-10

**Authors:** Ioana Elena Lile, Gianina Tapalagă, Diana Marian, Andra-Alexandra Stăncioiu, Christian Samoilă, Carolina Cojocariu

**Affiliations:** 1 Department of Dentistry, Faculty of Dentistry, “Vasile Goldis” Western University of Arad, 94-96 Revolutiei Blvd., 310025 Arad, Romania; lile.ioana@uvvg.ro (I.E.L.); cojocariu.carolina@uvvg.ro (C.C.); 2 Department of Odontotherapy and Endodontics, Faculty of Dental Medicine, “Victor Babes” University of Medicine and Pharmacy Timisoara, Eftimie Murgu Square 2, 300041 Timisoara, Romania; 3 “Dr. Samoilă Christian” Dental Office, 300208 Timişoara, Romania

**Keywords:** oral health, dietary interventions, oral hygiene, pediatric population, caries prevention, behavioural interventions, systematic review

## Abstract

**Background/Objectives:** Within pediatric healthcare systems, integrated strategies combining dietary and oral hygiene interventions have been proposed to improve oral health outcomes. In order to evaluate the effects of combined dietary and oral hygiene approaches on behavioral, knowledge-based, and clinical outcomes in pediatric populations, this systematic review synthesized evidence from intervention studies. **Methods:** A comprehensive literature search was conducted on 5 September 2025 using PubMed, Scopus, Web of Science, Google Scholar, and arXiv. Children and adolescents (0–18 years old) exposed to dietary and oral hygiene interventions with documented behavioral, clinical, or knowledge-related outcomes were included in eligible studies. Studies were stratified by age groups (0–5, 6–12, and 13–18 years) in order to address developmental heterogeneity. The Cochrane RoB 2 tool for randomized trials and the ROBINS-I tool for non-randomized studies with a comparator were used to evaluate the risk of bias. For single-arm pre–post studies, a narrative risk-of-bias assessment was conducted, as ROBINS-I is not designed for uncontrolled studies. Due to methodological and clinical heterogeneity, a narrative synthesis approach was applied. **Results:** Out of 1231 records, eight outcome-reporting intervention studies satisfied the inclusion criteria. Improvements in self-reported oral hygiene behaviors, dietary habits, and oral health knowledge were consistently linked to integrated interventions across developmental subgroups. There was little and inconsistent evidence for clinical outcomes, such as caries and periodontal indicators. **Conclusions:** In pediatric populations, integrated dietary and oral hygiene interventions seem to enhance behavioral and educational oral health outcomes. These findings highlight the need for developmentally tailored preventive strategies integrated within pediatric healthcare frameworks.

## 1. Introduction

With detrimental effects on quality of life, well-being, long-term health, and educational outcomes, dental caries and periodontal diseases continue to be among the most common chronic conditions affecting children and adolescents globally [1]. In addition to their clinical ramifications, these conditions pose a substantial public health and social burden, impacting healthcare resources, academic performance, school attendance, and general quality of life.

Conventional approaches to promoting oral health have often focused on single-component interventions, typically addressing dietary habits or oral hygiene practices in isolation. However, because oral diseases are multifactorial, prevention strategies may benefit from focusing on multiple modifiable risk factors simultaneously [2]. Dental caries and periodontal disease are primarily influenced by diet, especially when free sugars and highly processed foods are consumed frequently [3,4,5]. It is well known that free sugar consumption plays a major part in the development of dental caries and continues to be a key component of preventive measures. In addition, maintaining periodontal health and reducing dental plaque requires good oral hygiene [6]. Research indicates that, particularly in pediatric populations, dietary habits and dental hygiene practices are closely linked and impacted by behavioural and educational factors [7,8]. A comprehensive preventive strategy within pediatric healthcare involves integrated interventions that combine dietary counselling with oral hygiene education to improve oral health behaviours, knowledge, and potential clinical outcomes. These interventions are typically delivered in educational or community settings and aim to address multiple behavioural determinants simultaneously. However, because current research varies significantly in design, intensity, outcome measures, and follow-up duration, the efficacy of these integrated approaches remains unknown.

The adoption and maintenance of preventive oral health behaviors in pediatric populations may be influenced by behavioral and psychosocial factors in addition to dietary and oral hygiene practices. Studies have found that dental fear and phobia, stress, and mental health can all hurt the way people look after their teeth and how well they stick to treatment plans [9]. People who experience poor mental health often find their oral health standards to be low. As a result, tooth decay or gum disease can develop, further worsening the individual’s mental health state [10]. Various approaches, including motivational interviewing and educational interventions, help individuals improve their oral health. This can be achieved by tackling the reasons why people may not look after their teeth [11]. Healthcare professionals must be able to identify psychological disorders from the signs displayed by patients, because these could be signs of an underlying mental health problem [12]. Integrating psychological elements into dental care will improve treatment outcomes and overall patient well-being. Integrated interventions are based on established disease models, which have been extensively researched. It is the combination of host factors, fermentable carbohydrates in the diet, microbial plaque, and the passage of time that determines the occurrence of dental caries [13]. Systemic and behavioural factors interact in a complex way to shape periodontal health [14]. Although several risk factors (e.g., genetic susceptibility or tobacco exposure) are not readily modifiable, dietary patterns and oral hygiene practices represent key behavioural determinants that can be addressed through preventive and educational interventions [2,6,11].

Since dietary sugar exposure and insufficient plaque control share behavioural drivers, integrated approaches that address multiple behavioural determinants of oral diseases are increasingly recommended to promote pediatric oral health. In order to improve oral health behaviors and associated outcomes in children and adolescents, prior research has shown that multicomponent and behaviourally oriented strategies may be more effective than single-component interventions [2,11].

However, pediatric populations are a highly heterogeneous population, especially when it comes to biological development, behavioral autonomy, and eating habits. Early childhood, school-age children, and adolescents differ significantly in their oral hygiene habits, feeding habits, and nutritional exposures. Therefore, without proper stratification, evaluating integrated dietary and oral hygiene interventions across a wide age range may introduce bias and restrict the interpretability of results. In order to address this, the current review specifically takes developmental subgroups into account when choosing studies and interpreting data.

In order to investigate the effects of dietary and oral hygiene components on behavioral, knowledge-based, and clinical oral health outcomes in children and adolescents (0–18 years old), this systematic review sought to identify and synthesize evidence from intervention studies.

## 2. Materials and Methods

### 2.1. Protocol and Registration

This systematic review was conducted and reported in accordance with the PRISMA 2020 guidelines [15]. The review protocol was registered in the International Prospective Register of Systematic Reviews (PROSPERO; registration number CRD420261279079) prior to data extraction. The PRISMA checklist is provided in the Supplementary Materials.

### 2.2. Eligibility Criteria

Children and adolescents (0–18 years old) were included in primary empirical studies that qualified. Studies were divided into predetermined age subgroups: early childhood (0–5 years), middle childhood (6–12 years), and adolescence (13–18 years) in order to address developmental and nutritional heterogeneity. Studies with participants who were generally healthy were eligible; when the intervention focused on oral health behaviors, pertinent pediatric subgroups (such as teenagers wearing orthodontic appliances) were included. The effectiveness synthesis did not include studies that primarily focused on severe systemic disorders that could significantly change oral health behaviors. Subgroup-specific analyses were carried out whenever feasible, and results were interpreted within age-specific settings rather than as a single aggregated population due to the known change in food patterns, behavioral variables, and oral health risk profiles across developmental stages. The inclusion and exclusion criteria are presented in Table 1.

A structured preventive program that purposefully combined dietary modification and oral hygiene components within a coordinated framework to improve oral health outcomes was referred to as an “integrated intervention” for the purposes of this review. We differentiated among (i) additive approaches (components that coexist but are implemented independently), (ii) integrated interventions (components delivered within the same program but not explicitly linked to behavior), and (iii) behaviourally integrated interventions (components aligned within a unified behavior-change strategy targeting shared determinants, such as sugar exposure and biofilm control). Research that showed at least additive coordination of both elements within the same preventive framework was accepted. For inclusion, studies were required to report at least one oral hygiene component and one dietary component actively implemented within the same intervention framework.

### 2.3. Search Strategy

**Figure 1:**
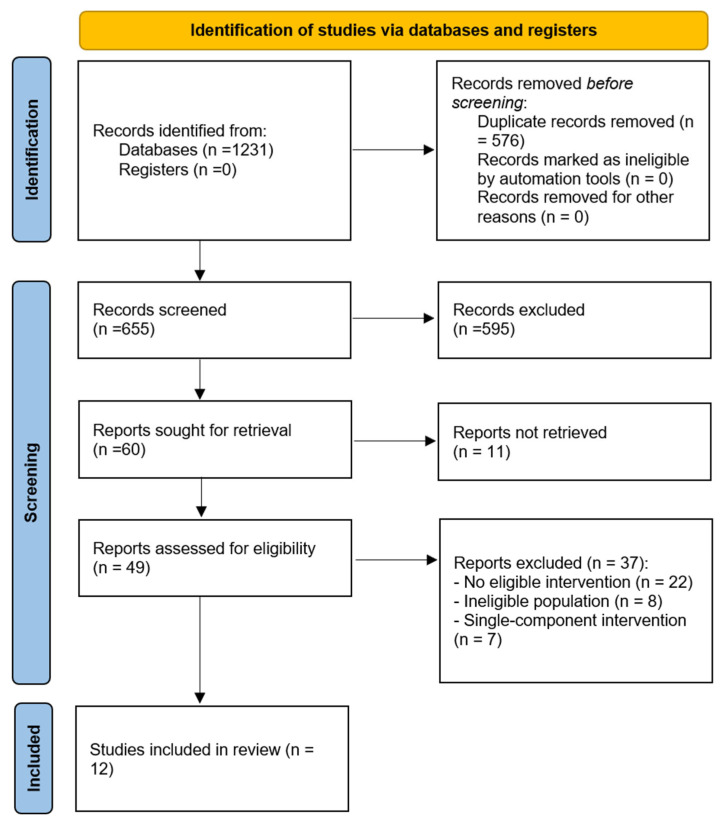
PRISMA 2020 flow diagram of the study selection process [15].

Each database search combined three main concepts using AND: Concept 1: oral health/oral hygiene/dental caries, Concept 2: diet/nutrition/sugar intake, Concept 3: children/adolescents/youth. Searches were limited to human studies involving participants aged 0–18 years. The studies were eligible regardless of location or setting type. Furthermore, no restrictions were placed on whether the studies were randomised, and the year range for each database entry was 2015–2025. To capture grey literature, such as study protocols and ongoing projects, we searched Google Scholar and arXiv. Unless a peer-reviewed full report with outcome data was available, non-peer-reviewed records and preprints were screened separately and excluded from the effectiveness synthesis. The search targeted PubMed, Scopus, Web of Science Core Collection, arXiv, and Google Scholar from 2015 to 2025 using controlled vocabulary and free-text terms related to oral hygiene, diet/nutrition, caries, periodontal health, and paediatric populations. Google Scholar and arXiv were used to identify relevant grey literature, such as protocols and preprints; all records from these sources were screened for study type, and duplicates were removed by comparing them with records retrieved from bibliographic databases. Targeted keywords were used across all relevant databases to systematically identify and retrieve literature relevant to the research area. Search terms included various combinations of: “oral hygiene,” “oral health,” “diet,” “nutrition,” “intervention,” “children,” “adolescents,” “school-based,” “community,” “behavioural change,” “caries prevention,” “periodontal health,” and related terms (Table 2). The study selection process was conducted in accordance with PRISMA 2020 guidelines and is illustrated in Figure 1 [15]. The full search strategies for all databases are provided in the Supplementary Materials.

### 2.4. Study Selection

A sequential screening procedure was used to choose the studies. Based on predetermined eligibility criteria, titles and abstracts were screened for relevance, considering both dietary and oral hygiene components. After that, the full texts of studies that might qualify were evaluated for inclusion. Any disputes were settled through dialogue. Screening and study selection were conducted independently by two reviewers. Discrepancies were resolved through discussion, and where necessary, a third reviewer was consulted.

### 2.5. Data Extraction

Research design, environment, and participant characteristics (such as initial oral health status and pertinent comorbidities/subgroups, like orthodontic treatment), sample size, components of the intervention, intervention duration, outcomes evaluated, and key findings were among the data extracted. A quantitative meta-analysis was not conducted due to significant clinical and methodological heterogeneity; instead, the results were narratively synthesized. Results were further analysed and interpreted by age subgroups (0–5, 6–12, and 13–18 years), intervention delivery mode, and follow-up duration in order to account for developmental heterogeneity. This stratified approach was used to reduce bias associated with aggregating populations with substantially different dietary behaviours and oral health determinants. Data extraction was performed independently by two reviewers using a standardized data extraction form. Disagreements were resolved by consensus. A third reviewer was consulted when consensus could not be reached.

### 2.6. Risk of Bias Assessment

Randomized controlled trials and cluster-randomized trials (Samuel et al. (2020) [16], Parihar et al. (2024) [17], Wu et al. (2021) [18], Aljafari et al. (2022) [19], and Meyer-Lueckel et al. (2016) [20]) were evaluated using the Cochrane Risk of Bias 2 (RoB 2) tool. The quasi-experimental study by Mohamed et al. (2024) [21] was assessed using the ROBINS-I tool. For single-arm pre–post studies (Ziari et al. (2023) [22] and Bolhaqueiro et al. (2024) [23]), a narrative risk-of-bias assessment was conducted, focusing on key domains such as confounding, outcome measurement, and missing data, as ROBINS-I is not designed for uncontrolled studies. These tools examine domains including confounding, participant selection, classification of interventions, deviations from intended interventions, missing data, outcome measurement, and selective reporting. Interpretation of effectiveness signals was guided by study-level risk-of-bias judgments. Mohamed et al. (2024) [21] and Ziari et al. (2023) [22] were judged to have a moderate-to-high risk of bias due to quasi-experimental designs and potential confounding, while Bolhaqueiro et al. (2024) [23] raised concerns about incomplete outcome reporting. Graphical risk-of-bias summaries were generated to enhance transparency and facilitate cross-study comparison. Due to the limited number and heterogeneity of included studies, formal assessment of reporting bias (e.g., funnel plots) was not performed. A formal GRADE assessment was not conducted due to substantial heterogeneity across studies.

## 3. Results

### 3.1. Study Selection

Database and other source searches yielded 1231 records in total. 655 records remained for title and abstract screening after 576 duplicates were removed. Based on predetermined eligibility criteria, 595 of these records were eliminated. Eleven of the sixty reports requested for retrieval were unsuccessful. 49 full-text articles were evaluated for eligibility. 37 studies were eliminated after full-text review (22 because there was no eligible intervention, 8 because the population was ineligible, and 7 because the interventions were single-component). The inclusion criteria were satisfied by twelve studies. Eight of these were outcome-reporting intervention studies that were part of the effectiveness synthesis, and four protocol studies were kept for contextual mapping. The PRISMA 2020 flow diagram (Figure 1) shows the study selection procedure [15].

### 3.2. Study Characteristics

To maintain transparency, the studies are divided into two groups: (i) protocol/implementation reports kept for contextual mapping purposes only (*n* = 4; reported in Supplementary Table S1), and (ii) outcome-reporting intervention studies that were part of the effectiveness synthesis (*n* = 8). A unique behavioural and clinical subgroup of adolescents with fixed orthodontic appliances was included in one study, but most outcome-reporting studies focused on generally healthy school- or preschool-aged children. Table 3 presents an organized summary of the target age groups and the methods of intervention delivery.

In order to account for variations in dietary practices, behavioral autonomy, and intervention responsiveness, results were interpreted within developmental subgroups rather than as a single pooled population.

#### 3.2.1. Outcome-Reporting Intervention Studies

Randomized controlled trials, cluster-randomized trials, quasi-experimental studies, and pre-post intervention studies involving preschoolers, school-aged children, adolescents, and particular pediatric subgroups were among the outcome-reporting studies that were part of the effectiveness synthesis. These studies reported behavioral, knowledge-based, and/or clinical oral health outcomes and assessed integrated dietary and oral hygiene interventions.

In a recent Indian randomized controlled study, Samuel et al. (2020) assessed a multicomponent school-based intervention over a two-year period with 420 preschoolers between the ages of three and five [16]. The intervention includes oral health education, a school policy limiting the intake of sugary snacks, and daily toothbrushing under supervision using fluoridated toothpaste [16].

In 2023, Ziari et al. conducted a single-arm pre-post design trial in Iran on 30 teenagers aged 12–16 years with fixed orthodontic appliances. Participants took part in MI sessions. These focused on reducing sugary snack intake and on proper brushing and interdental cleaning [22].

Researchers in India conducted a six-month study involving 5000 schoolchildren aged 10 to 15. The study was a cluster-randomised controlled trial in which the children received oral hygiene education [17].

A quasi-experimental study, conducted by Mohamed et al. (2024) in Egypt, involved 400 primary school children aged 11–12 years [21]. These children participated in a nurse-led educational program. The programme provided them with information on dietary choices for oral health, as well as the best oral hygiene practices. Oral health knowledge, attitudes, and self-reported hygiene practices all significantly improved following the intervention [21].

Researchers in Portugal conducted a study in 2024 to evaluate the impact of the community-based initiative known as SORRISOS. This initiative, targeted at 157 Portuguese children aged 3 to 6, included activities to enhance oral literacy, promote proper tooth-brushing techniques, and provide nutrition guidance to children and their families [23].

Researchers in Hong Kong conducted a trial involving adolescents to assess the benefits of motivational interviewing in reducing junk food consumption and improving toothbrushing [18].

Researchers in Jordan developed a game for children to promote good oral hygiene and healthy eating habits. The game was tested among school children. It educated the children on how to look after their teeth and eat a healthy diet [19].

In a German study by Meyer-Lueckel et al. (2016), resin infiltration therapy was the main intervention being studied, and each participant received personalized oral hygiene instructions and non-cariogenic dietary counseling [20]. The study was included because food and oral hygiene components were part of the preventative framework; however, there is no direct correlation between these behavioral components and the reported clinical outcomes [20].

#### 3.2.2. Protocol and Implementation Studies

The following studies are protocol or implementation reports and did not contribute outcome data to the effectiveness synthesis. They are presented for contextual mapping only and detailed in Supplementary Table S1.

The protocol and implementation studies that combined oral health education with nutrition or more general health promotion are described in this subsection; however, they did not provide outcome data for the effectiveness synthesis.

Gao et al. (2015) released the protocol of a randomized controlled trial assessing the effectiveness of motivational interviewing and oral health education in enhancing adolescent oral health behaviors [24].

An Indonesian community programme based in a primary school delivered oral health education and optimised nutrition advice together [25].

A cluster-randomised trial conducted in rural Uganda by Muhoozi et al. (2018) reported on the follow-up of nutrition and toddler hygiene education [26].

Gao et al. (2015) reported on a school health education project in Hong Kong [24]. The trial tested a health education programme aimed at improving oral hygiene by altering tooth-brushing and dietary patterns [24].

Supplementary Table S1 provides contextual insight into the development and structure of integrated interventions.

### 3.3. Risk of Bias Assessment

**Figure 2:**
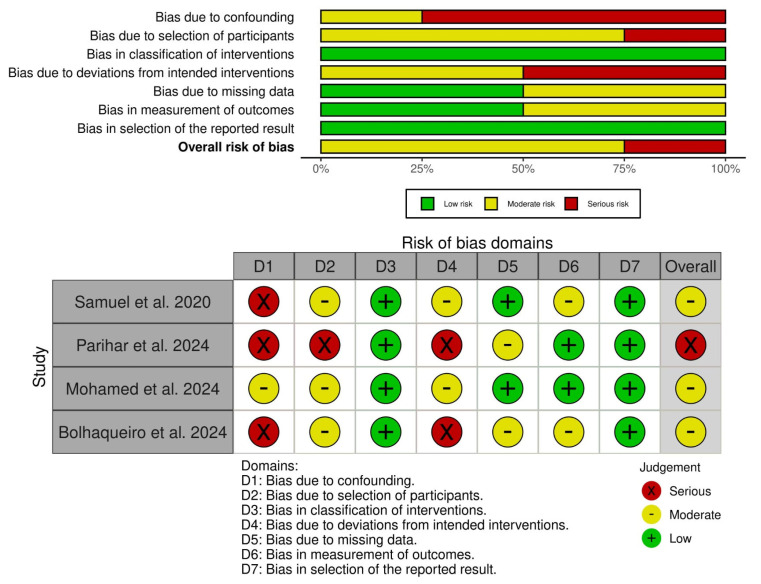
Risk-of-bias traffic light plot for non-randomized studies assessed using the ROBINS-I tool (green = low risk, yellow = moderate risk, orange = serious risk, red = critical risk) [16,17,21,23].

**Figure 3:**
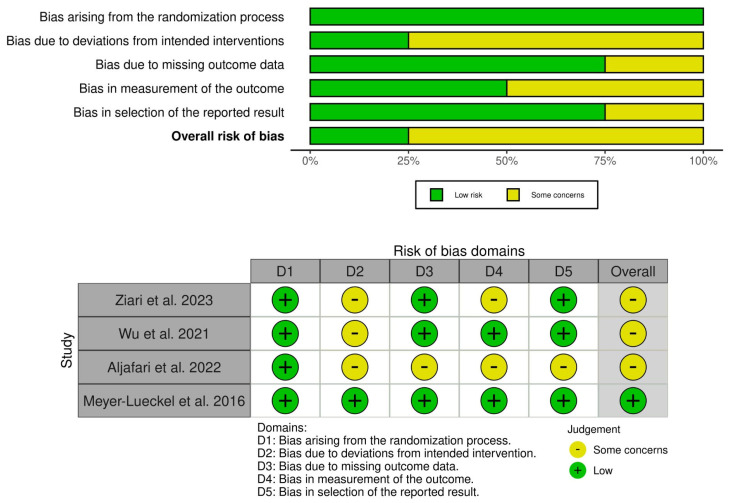
Risk-of-bias summary for randomized controlled trials assessed using the Cochrane RoB 2 tool. Color coding follows standard RoB 2 judgment categories [18,19,20,22].

The Cochrane Risk of Bias 2 (RoB 2) tool was used for randomized controlled trials, and the ROBINS-I tool was used for non-randomized studies with a comparator. Single-arm pre–post studies were assessed narratively, as ROBINS-I is not designed for uncontrolled studies. The risk of bias was assessed using design-appropriate instruments. Three of the randomized studies that were evaluated using the RoB 2 tool were categorized as having some concerns, while one study was deemed to be at low risk of bias. No randomized study was deemed to be highly biased. One study was categorized as low risk, two as moderate risk, and one as serious risk of bias among the non-randomized studies evaluated using ROBINS-I. Although there were some issues with incomplete outcome reporting, outcome measurement, and deviations from planned interventions, the overall risk-of-bias profile indicates that the available data should be interpreted cautiously. The assessments are summarized graphically in Figure 2 and Figure 3. The Risk-of-bias VISualization (robvis) tool was used to create the transparent graphical summaries shown in Figure 2 and Figure 3 [27].

### 3.4. Synthesis of Findings

The included studies suggest that a combination of oral hygiene and dietary interventions can positively affect children’s and adolescents’ knowledge of oral health, behaviour, and clinical signs such as dental caries and periodontal status (Table 4).

Sample sizes varied widely across studies, from small single-arm interventions to large cluster-randomized trials involving several thousand participants. The participants’ ages, reflecting developmental heterogeneity, ranged from early preschool (3–5 years) to mid-adolescence (up to 16 years). Intervention lengths ranged widely from short programs lasting a few weeks to lengthy follow-up periods of up to several years, as indicated in Table 4. Studies’ reports of the gender distribution varied, but available samples typically contained both male and female participants without significant imbalance. Nevertheless, not every study offered comprehensive data that was broken down by sex. Clinical outcomes were objectively assessed using validated indices (e.g., dmft/DMFT, Plaque Index, Gingival Index, CPI, radiographic lesion progression), whereas behavioural and knowledge outcomes were predominantly self-reported or questionnaire-based. A variety of oral hygiene interventions were included in the study, the most commonly used being supervised toothbrushing with fluoride toothpaste, oral hygiene instruction, an oral hygiene demonstration, and teaching on plaque prevention. Interventions incorporating motivational oral hygiene behaviours also included education on oral health and instruction on proper toothbrushing.

Several components of the diet incorporated various strategies: policies limiting sugary snacks at school or in the programme, nutrition education, advice on healthy eating tailored to the individual, dental health, and personal counselling. Interventions promoted a healthy diet in general and used motivational interviewing techniques to encourage positive dietary changes. They also educated the participants about the differences between foods that cause tooth decay and those that do not.

### 3.5. Outcome Measures and Results

To enable clear synthesis and interpretation across diverse study designs, results are presented independently as clinical, behavioural, and knowledge-based findings.

Additional interpretations of the results are made based on (i) age group (preschool, school-age, and adolescents), (ii) delivery method (supervised practice versus counseling/motivational interviewing versus digital/game-based), and (iii) follow-up duration in order to examine heterogeneity.

Outcomes were heterogeneous and included objectively assessed clinical indices (e.g., caries increment, plaque/gingival indices) as well as self-reported behaviours and questionnaire-based knowledge/attitude measures.

#### 3.5.1. Clinical Outcomes

Only a small subset of studies reported clinical outcomes pertaining to periodontal health and caries incidence/progression. dmft/DMFT indices, radiographic lesion progression, plaque, gingival, and community periodontal indices were among the objective measures. After two years, one longer trial found that the intervention group’s mean caries increment was lower, but other studies found that periodontal indices (e.g., plaque/gingival indices) had changed during a shorter period. Given variations in design, baseline risk, and risk-of-bias profiles, these results should be regarded as early indicators rather than conclusive proof of clinical efficacy.

Table 5 presents a structured summary of the clinical outcomes reported in all included studies.

Specifically for periodontal health, Parihar et al. [17] reported significant reductions in the Community Periodontal Index, Gingival Index, and Plaque Index with a multicomponent school-based program. The mean plaque index decreased from 0.99 ± 0.43 to 0.37 ± 0.16 following motivational interviewing. The gingival index was also reduced from 0.99 ± 0.56 to 0.30 ± 0.20.

Table 6 summarizes the knowledge-based and behavioral outcomes from all of the included studies, showing steady gains in self-reported oral hygiene habits, dietary habits, and oral health awareness.

#### 3.5.2. Behavioural Outcomes

The programme, which included a dental health education component, led to noticeable improvements in the subjects’ oral hygiene behaviour. Individuals in the intervention groups showed considerable improvement in their toothbrushing habits, specifically an increase in brushing frequency and quality. Oral health awareness and perceptions were also enhanced. Compliance with oral hygiene recommendations was better than in the control group. Research indicates that combining behavioural education with a skill-based oral hygiene education programme is effective in promoting daily oral hygiene practices. Several studies documented improvements in diet-related behaviour, with multiple interventions reporting that participants ate fewer sugary snacks and were more aware of the link between diet and oral health. Those who took part showed a better understanding of which foods can cause tooth decay than those who could not, thereby suggesting that the lessons about diet had led to improved dietary choices.

#### 3.5.3. Knowledge and Attitudes

Significant improvements in oral health knowledge scores have been observed following interventions involving oral hygiene education and dietary advice. Not only were there improvements in how people thought about keeping their mouths clean and maintaining a balanced diet, but also in how they reported adopting healthier habits. Various educational and parental assessments showed a significant improvement in the behaviour of children who took part in the project.

## 4. Discussion

A quantitative meta-analysis was not possible due to significant heterogeneity across study designs, populations, intervention components, outcome measures, and follow-up durations. As a result, rather than estimating the relative efficacy of various intervention types, a narrative synthesis was conducted to find recurring patterns among the included studies [16,17,18,19,20,21,22,23]. Specifically, meaningful quantitative pooling of the data was not possible due to the small number of studies reporting similar clinical outcomes, such as periodontal indices and dmft/DMFT.

Table 3 and Table 4 provide a summary of the descriptive comparison of intervention duration, delivery mode, intensity, and follow-up periods in order to examine heterogeneity across studies.

Different patterns were observed by mode of delivery and age (Table 3). Programs that included structured school policies (e.g., supervised toothbrushing) were implemented in preschools, and younger school-age populations (e.g., limits on sugary snacks) tended to report more consistent behavioural improvements and, in a few instances, positive clinical signals over an extended period. Counselling-based interventions were more prevalent in adolescents (e.g., motivational interviewing), and the results were primarily self-reported and behavioural, with little to no clinical follow-up. Because of the variations in design and measurement, these observations should be regarded as exploratory.

Overall, the most consistent improvements in oral hygiene behaviors and, in certain studies, positive clinical outcomes seemed to come from supervised school-based interventions used with preschoolers and school-age children. Studies with longer follow-up periods were more likely to report quantifiable clinical effects, but behavioral and educational benefits were seen across a wide range of intervention durations. On the other hand, there is little evidence of long-term clinical outcomes from counseling-based and motivational interviewing approaches, which were more commonly used with adolescents. These approaches mainly improved self-reported behaviors and dietary habits. While there was still little proof of clinical benefits, digital and game-based interventions seemed to be especially successful in raising knowledge and engagement.

These results align with the review’s main goal, which is to assess how integrated dietary and oral hygiene interventions affect behavioral, knowledge-based, and clinical outcome domains. The evidence supporting clinical benefits is still sparse and inconsistent, despite the fact that improvements were most consistently seen for behavioral and educational outcomes.

Overall, data from the included intervention studies indicate that integrated dietary and oral hygiene programs are associated with improvements in children’s and adolescents’ self-reported behaviours, attitudes, and knowledge of oral health [28,29,30].

These results are consistent with earlier research indicating that multicomponent preventive strategies that target behavioral and environmental factors may result in longer-lasting gains in pediatric populations’ oral health [30,31,32].

The most frequently reported benefits in both school- and community-based settings were behavioural outcomes, such as improved brushing habits and reduced consumption of sugary foods [16,17,18,19,21,22,23]. On the other hand, there was little and inconsistent evidence for clinical outcomes such as improved periodontal health and reduced caries. This variability in clinical outcomes is often influenced by the materials and techniques used in current practice [31]. Positive clinical changes were reported in some studies, but these findings were based on a limited number of trials with varying designs and follow-up periods. Another thing to take into account is that a number of interventions combined the use of fluoridated toothpaste or other fluoride-based preventive measures with oral hygiene education and supervised brushing. As a result, it is difficult to distinguish between the independent effects of fluoride exposure and behavioral oral hygiene practices on clinical outcomes based on the evidence currently available.

As a result, conclusions about clinical efficacy remain provisional and should be interpreted with caution [16,17,18,22]. Longer-term behavioural improvements seemed to be linked to interventions administered at younger ages, particularly considering the morphological development patterns observed in paediatric populations [33,34].

While interactive or counselling-based approaches were more commonly employed in adolescent populations, structured and supervised programs were more frequently associated with positive outcomes in younger children. Rather than drawing firm comparative conclusions, these observations show patterns within the included studies [16,17,18,19,21,22,23]. It is important to recognise significant limitations. Comparability was hampered, and quantitative synthesis was impossible due to heterogeneity in interventions and outcomes.

The inclusion of a wide pediatric age range presents one of the review’s primary methodological challenges. This introduces heterogeneity in dietary exposure, behavioral patterns, and oral health risk even though it represents practical preventive strategies. The results were interpreted within age-specific subgroups to mitigate this limitation. To increase comparability and bolster causal inference, future systematic reviews might profit from concentrating on more specific developmental categories.

The social, educational, and cultural contexts of the included populations differ, and there is a great deal of variation in the intervention’s intensity, implementation techniques, and educational goals. A number of programs were exploratory in nature, which makes it more difficult to determine the best intervention model and restricts direct comparisons between studies.

Numerous studies had non-randomised designs, small sample sizes, or brief follow-up periods, which increased uncertainty and increased susceptibility to bias [16,17,18,19,20,21,22,23]. The strength and interpretability of the available evidence are significantly diminished by these limitations, especially when it comes to clinical outcomes.

Consequently, results from quasi-experimental and single-arm studies are regarded as preliminary and hypothesis-generating, whereas randomised and cluster-randomised trials are given more weight in interpretation.

Well-designed trials with more extended follow-up periods and standardised intervention components should be given priority in future research. The creation of core outcome sets would strengthen future evidence synthesis in this area and enhance comparability [32,35,36].

Dietary free sugars are thought to be the main etiological factor in dental caries development, which is in line with Sheiham’s findings. This emphasizes the critical role that dietary interventions play in comprehensive caries prevention strategies, in addition to proper oral hygiene practices [37].

Since smoking behavior, a known risk factor for adolescent periodontal disease, was not routinely evaluated in the included studies, it was not possible to analyze it in this review. Incorporating tobacco-related behavioural components into future preventive interventions aimed at older pediatric populations may prove advantageous.

Because study designs, intervention features, and outcome measures varied widely, a formal GRADE certainty assessment was not conducted. However, because of methodological flaws, variations in follow-up length, and the prevalence of behavioral outcomes, the overall strength of the available evidence should be considered limited to moderate.

The review aimed to provide an evidence-informed synthesis of recent findings rather than formal clinical recommendations due to the heterogeneity of the available evidence.

From a healthcare perspective, these findings support the integration of preventive oral health strategies within existing pediatric care pathways.

There are still significant gaps in the current literature, despite the positive results. Direct comparisons between the included studies were limited by significant heterogeneity in intervention designs, outcome measures, and follow-up duration. Additionally, only a small number of studies reported long-term clinical outcomes like periodontal indicators, dmft/DMFT scores, or caries incidence. Future studies should concentrate on carefully planned randomized controlled trials with extended follow-up periods and standardized outcome measures. It would also be possible to determine which integrated preventive strategies work best for particular pediatric populations by conducting comparative studies that assess these strategies across various developmental stages.

## 5. Conclusions

The evidence from eight outcome-reporting intervention studies assessing integrated dietary and oral hygiene programs for kids and teenagers was compiled in this systematic review. The evidence that is currently available shows that, especially in school-based settings, these integrated interventions are consistently linked to improvements in oral health-related knowledge, attitudes, and self-reported behaviors. A small number of diverse studies with different designs, intervention intensities, and follow-up times produced the reported clinical benefits. Because of this, it is impossible to draw firm conclusions about clinical efficacy from the available data. Oral hygiene and dietary interventions seem promising overall for encouraging positive oral health behaviors in pediatric populations. Clinical recommendations that alter practice regarding caries or periodontal outcomes are not supported by sufficient evidence at this time. To better evaluate efficacy and sustainability, future research should give priority to well-designed, sufficiently powered studies with longer follow-up times, standardized intervention components, and precisely defined clinical and behavioural outcome measures. To improve the validity and interpretability of future studies on integrated oral health interventions in pediatric populations, stratified approaches are crucial. Integration within existing pediatric healthcare delivery models may represent a key step toward scalable and sustainable prevention strategies.

## Figures and Tables

**Table 1:** Criteria of article selection.

Inclusion Criteria	Exclusion Criteria
Population: Children and adolescents (aged 0–18 years)	Adults, over the age of 19
Intervention: Studies implementing interventions that combined both oral hygiene and dietary components	Studies implementing interventions exclusively about oral hygiene or exclusively about dietary components
Oral hygiene components (toothbrushing education, plaque control, fluoride use, oral health education)	without oral hygiene components
Dietary components (nutrition education, sugar reduction, dietary counseling, healthy eating promotion)	Animal or in vitro study
Primary Outcomes: Caries prevention, periodontal health measures, dietary compliance	Conference abstracts, letters, commentaries
Secondary Outcomes: Knowledge, attitudes, behavioural changes, general health indicators	
Study Design: Randomized controlled trials, cluster-randomized trials, quasi-experimental studies, controlled before-and-after studies, and community program evaluations Publication period: 2015–2025	Published outside 2015–2025

**Table 2:** Strings, limits, and dates of search strategies.

Databases	Search Strategies	Nr. of Articles Found	Filters:
**PubMed**	(“oral health”[Mesh] OR “oral hygiene”[Mesh] OR “dental caries”[Mesh] OR “oral health”[tiab] OR “oral hygiene”[tiab] OR “toothbrushing”[tiab] OR “tooth brushing”[tiab] OR “plaque index”[tiab] OR “gingival index”[tiab]) AND (“diet”[Mesh] OR “diet, cariogenic”[Mesh] OR “dietary sugars”[Mesh] OR diet*[tiab] OR nutrition*[tiab] OR “sugar intake”[tiab] OR “sugary snack*”[tiab] OR “sugar-sweetened”[tiab]) AND (“Child”[Mesh] OR “Adolescent”[Mesh] OR child*[tiab] OR adolescent*[tiab] OR teen*[tiab] OR youth[tiab] OR “schoolchild*”[tiab] OR preschool*[tiab])	1000	Limits: Humans; Age: child and adolescent (0–18 years); Publication type: clinical trials, cross-sectional studies, case series or reports, case–control studies, clinical trials, and controlled trials. Up to 5 September 2025
**Scopus**	TITLE-ABS-KEY (“oral health” OR “oral hygiene” OR “dental caries” OR toothbrushing OR “tooth brushing” OR “plaque index” OR “gingival index”) AND TITLE-ABS-KEY (diet* OR nutrition* OR “sugar intake” OR “sugary snack*” OR “sugar-sweetened”) AND TITLE-ABS-KEY (child* OR adolescent* OR teen* OR youth OR schoolchild* OR preschool*)	21	Limits: Document type = article; Subject area including medicine/dentistry/health; Humans; Timespan up to 5 September 2025
**Web of Science Core Collection**	TS = (“oral health” OR “oral hygiene” OR “dental caries” OR toothbrushing OR “tooth brushing” OR “plaque index” OR “gingival index”) AND (diet* OR nutrition* OR “sugar intake” OR “sugary snack*” OR “sugar-sweetened”) AND (child* OR adolescent* OR teen* OR youth OR schoolchild* OR preschool*)	40	Limits: Document type = Article; Research areas including Dentistry OR Public, Environmental & Occupational Health OR Pediatrics OR Nutrition & Dietetics; Timespan up to 5 September 2025.
**arXiv**	(“oral health” OR “oral hygiene” OR “dental caries” OR toothbrushing OR “tooth brushing”) AND (diet OR nutrition OR “sugar intake” OR “sugary snack” OR “sugar-sweetened”) AND (child OR children OR adolescent OR adolescents OR youth OR school OR preschool)	20	Subject categories likely to contain health/behavioural research (e.g., q-bio, stat. AP, cs.HC); date up to 5 September 2025.
**Google Scholar**	First pass (broad): “oral health” “oral hygiene” diet OR nutrition “children” OR adolescents’ caries intervention	150	Since 2015.

**Table 3:** Target age groups, environments, and methods of delivery of the outcome-reporting studies that are included.

Study	Age Group (Years)	Setting	Delivery Mode	Notes
Samuel et al. 2020 [16]	3–5	Preschool/school-based	Supervised daily brushing + school programme	Policy element (sugary snacks restricted)
Ziari et al. 2023 [22]	12–16	Clinical (orthodontic)	Individual MI + skills training	Pre–post design
Parihar et al. 2024 [17]	10–15	School-based	School programme + supervised practice	Cluster-RCT
Mohamed et al. 2024 [21]	11–12	School-based	Nurse-led education	Quasi-experimental
Bolhaqueiro et al. 2024 [23]	3–6	Community-based	Literacy activities + counselling	Family involvement
Wu et al. 2021 [18]	Adolescents	School/community	MI (individual/group)	RCT; longer follow-up
Aljafari et al. 2022 [19]	Primary school	School-based	Digital game-based education	RCT
Meyer-Lueckel et al. 2016 [20]	Adolescents/young people	Clinical	Individual counselling + clinical treatment	Pragmatic RCT

**Table 4:** Characteristics and primary outcomes of the included studies.

Study	Participants (n)	Intervention Length/Follow-Up	Oral Hygiene Components	Dietary Components	Outcomes (Type of Assessment)	Key Findings	Main Limitation
Samuel et al. 2020 [16]	420	2 years	School-based oral health education, teacher-supervised toothbrushing, the use of fluoride toothpaste, regular dental check-ups, and a dental sealant programme have been implemented.	School policy prohibiting sugary snacks	Caries increment (dmft/DMFT index) [O]; Plaque Index [O]; Gingival Index [O]	20% absolute caries risk reduction; mean increment 0.4 vs. 0.9	Multicomponent intervention; contribution of individual components cannot be isolated
Ziari et al. 2023 [22]	30	6 weeks	Individual MI counselling; brushing/flossing technique training	MI addressing sugary snack consumption	Plaque Index [O]; Gingival Index [O]; Brushing behaviour [SR]; Sugary snack intake [SR]	Periodontal indices pre-treatment: Plaque index 0.99, Gingival index 0.99; Post-treatment: Plaque index 0.37, Gingival index 0.30.	Small sample size and absence of a control group
Parihar et al. 2024 [17]	5000	6 months	Oral hygiene instruction; supervised practice; dental checkups	Dietary counselling included in program	Community Periodontal Index (CPI) [O]; Gingival Index [O]; Plaque Index [O]; Knowledge [Q]; Oral hygiene practices [SR]	Significant reductions in CPI, Gingival Index, and Plaque Index; exact effect estimates not reported.	Potential risk of bias related to study design
Mohamed et al. 2024 [21]	400	3 months	Delivering dental health information, oral hygiene instruction, and interdental cleaning instruction.	Education on dietary choices promoting dental health	Oral health knowledge [Q]; Attitudes [Q]; Hygiene practices [SR]	Significant improvements in oral health knowledge, attitudes, and self-reported hygiene practices (effect sizes not reported).	Quasi-experimental design
Bolhaqueiro et al. 2024 [23]	157	6 months	Oral health literacy promotion; hygiene skill development; clinical examinations	Nutrition counselling; breastfeeding support	Clinical oral examination [O]; Behavioural survey [SR]	70% of participants presented oral diseases; beneficial behavioural changes were reported, although quantitative effect estimates were not available.	Limited outcome quantification
Wu et al. 2021 [18]	304	24 months follow-up	Motivational interviewing for toothbrushing behaviour	MI for snack intake reduction	Caries outcomes (DMFT/cavitated lesions) [O]; Brushing behaviour [SR]; Sugary snack intake [SR]	Fewer cavitated teeth were observed in the MI groups that reduced sugar intake; exact effect estimates were not reported.	Predominantly behavioural outcomes
Aljafari et al. 2022 [19]	109	8 weeks	Video game-based toothbrushing education	Video game dietary knowledge education	Plaque score [O]; Dietary knowledge [Q]; Oral hygiene behaviours [SR]	Improved dietary knowledge, whereas no statistically significant changes in plaque scores were observed.	Short follow-up period
Meyer-Lueckel et al. 2016 [20]	121	3 years follow-up	Individualized oral hygiene instructions; flossing guidance	Non-cariogenic diet instructions for all participants	Radiographic lesion progression [O]	Resin infiltration was associated with a decrease in lesion progression; however, all participants received dietary counseling and oral hygiene, making it impossible to pinpoint the precise role of the behavioral components.	Clinical effect primarily associated with resin infiltration

[O] = objectively assessed clinical outcome; [SR] = self-reported behaviour; [Q] = questionnaire-based knowledge/attitudes.

**Table 5:** Summary of clinical outcomes reported in included studies.

Study	Clinical Outcome	Measure/Index	Direction of Effect
Samuel et al. 2020 [16]	Caries increment	dmft/DMFT	Reduced
Ziari et al. 2023 [22]	Periodontal indices	Plaque Index, Gingival Index	Improved
Parihar et al. 2024 [17]	Periodontal indices	CPI, Plaque Index, Gingival Index	Improved
Bolhaqueiro et al. 2024 [23]	Oral disease prevalence	Clinical examination	Mixed
Wu et al. 2021 [18]	Caries incidence	DMFT/cavitated lesions	Reduced
Aljafari et al. 2022 [19]	Plaque accumulation	Plaque score	No significant change
Meyer-Lueckel et al. 2016 [20]	Lesion progression	Radiographic assessment	Slowed progression

**Table 6:** An overview of the knowledge-based and behavioral outcomes documented in the included studies.

Study	Behavioural Outcomes	Knowledge/Attitude Outcomes	Direction of Effect
Ziari et al. 2023 [22]	Improved brushing behaviour; reduced sugary snack intake	Not reported	Improved
Parihar et al. 2024 [17]	Improved oral hygiene practices	Increased oral health knowledge	Improved
Mohamed et al. 2024 [21]	Improved hygiene practices	Increased knowledge and positive attitudes	Improved
Bolhaqueiro et al. 2024 [23]	Behavioural changes reported	Not clearly quantified	Mixed improvement
Wu et al. 2021 [18]	Improved brushing frequency; reduced sugar intake	Not reported	Improved
Aljafari et al. 2022 [19]	Improved oral hygiene behaviours	Increased dietary knowledge	Improved
Samuel et al. 2020 [16]	Supervised brushing; school policy restricting sugary snacks	Not reported	Improved
Meyer-Lueckel et al. 2016 [20]	Dietary counselling + OHI provided to all	Not reported	Not reported

## Data Availability

No new data were created or analyzed in this study. Data sharing is not applicable to this article.

## Supplementary Materials

The following supporting information can be downloaded at: https://www.mdpi.com/article/10.3390/healthcare14142074/s1, Table S1. Protocol and implementation studies included for contextual mapping (not contributing outcome data).
