# Supplementary material for: Developmentally Stratified Integrated Preventive Strategies for Pediatric Oral Health: A Systematic Review with Healthcare Implications
Source: Healthcare (Basel). 2026 Jul 10;14(14):2074. doi: 10.3390/healthcare14142074 (PMC13411734; doi:10.3390/healthcare14142074)
Supplement: Supplementary file 1 [file healthcare-14-02074-s001.zip › healthcare-4251761-supplementary.pdf]

Table S1. Protocol and implementation studies included for contextual mapping (not contributing outcome data).

Study	Setting	Population	Intervention focus	Status
Wertheim & Miller et al. 2016	After-school program (USA)	School-aged children/adolescents	Oral health + nutrition education	Protocol
Dewi et al. 2024	Primary school (Indonesia)	Primary school children	Oral health education + nutrition	Implementation report
Muhoozi et al. 2018	Rural Uganda	Toddlers	Hygiene + nutrition education	Incomplete outcome reporting
Gao et al. 2015	School-based (Hong Kong)	Schoolchildren	Oral hygiene + dietary counselling	Protocol
